# Strength of Partially Encased Steel-Concrete Composite Column for Modular Building Structures

**DOI:** 10.3390/ma15176045

**Published:** 2022-09-01

**Authors:** Keum-Sung Park, Sang-Sup Lee, Kyu-Woong Bae, Jiho Moon

**Affiliations:** 1 Advanced Building Research Division, Korea Institute of Construction Technology (KICT), Goyang-si 10223, Gyeonggi-do, Korea; 2 Department of Civil Engineering, Kangwon National University, Chuncheon-si 24341, Gangwon-do, Korea

**Keywords:** steel-concrete composite structure, modular structural system, nonsymmetrical composite column, column buckling, *P*–*M* interaction curve

## Abstract

Modular structural systems have been used increasingly for low- and mid-rise structures such as schools and apartment buildings, and applications are extending to high-rise buildings. To provide sufficient resistance and economical construction of the high-rise modular structural system, the steel-concrete composite unit modular structure was proposed. The proposed composite unit modular system consists of the composite beam and the partially encased nonsymmetrical composite column. The outside steel member of the composite column has an open section, and is manufactured using a pressed forming procedure so that easy joining connecting work and manufacturing cost reductions are possible. However, the design methods are complicated due to the inherent nonsymmetrical properties of the section. Therefore, in this study, the focus was made on the strength evaluation and development of design methods for the partially encased nonsymmetrical steel-concrete composite column. Four full-scale specimens were constructed and tested. The experimental study focused on the effect of the slenderness ratio of the column, eccentricity, and the through bars on the strength of such columns. Additionally, the *P*–*M* interaction curve to estimate the strength of the proposed composite column under general combined loading was developed based on the plastic stress distribution method. The results indicate that the through bars are needed to delay the local buckling and distribute the loading uniformly throughout the composite column. Finally, the proposed design methods provide a conservative strength prediction of the proposed composite column.

## 1. Introduction

The modular structural system consists of repetitive-unit modular structures which are assembled to complete the building [1]. The applications of the modular structural system are increasing for low- and mid-rise buildings, such as those for schools and apartments. A mid-rise modular apartment was successfully constructed in South Korea as a demonstration of the modular structural system.

Several studies on the modular structural system have been conducted in the last decade [1,2,3,4,5,6,7,8,9,10,11,12,13,14,15]. In these studies, lightweight-module systems for high-rise building applications and easy joining connections were major research subjects. The steel structure is generally used for modular construction. However, reinforced concrete or steel-concrete composite materials are more suitable for high-rise building applications due to their enhanced stiffness and resistance [4,7]. Furthermore, the composite construction can provide excellent fire resistance compared to an ordinary steel structure [16].

**Figure 1:**
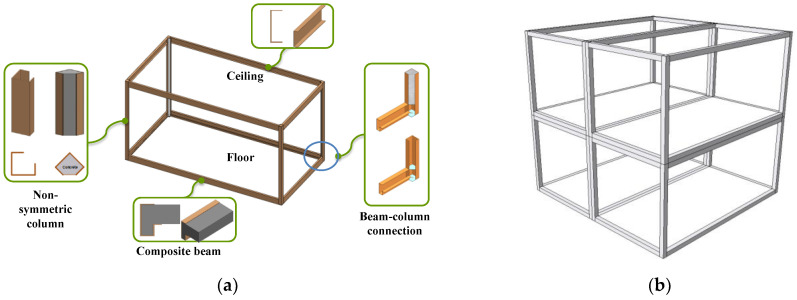
The proposed composite modular unit structure: (**a**) components; (**b**) assembly of units.

**Figure 2:**
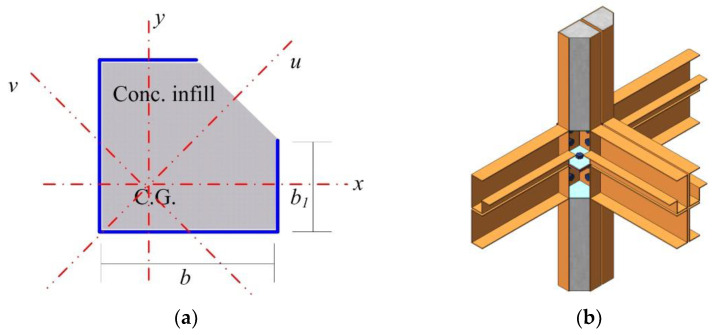
Nonsymmetrical composite column: (**a**) cross section; (**b**) connection between modules.

Corner-supported modules are often used for the modular structural system. For this system, the external loads are transferred through the edge beams and column of the module [3,4]. A new type of composite corner-supported module was developed by the authors, as shown in Figure 1. The components of the proposed composite corner-supported modular unit are shown in Figure 1a. This module consists of a partially encased nonsymmetrical composite column, a composite beam, a floor, and a ceiling. These units can be assembled as shown in Figure 1b. In the case of the composite column, a nonsymmetrical section shown in Figure 2a was adopted for the following reasons. The outside steel member was manufactured using a pressed forming procedure so that the manufacturing cost was less than the cost of using a rolled member. Easy and robust joining connection is very important for modular construction and several studies have been conducted to improve the joint of the modules [11,12,13,14,15]. In this study, an open section is used for the outside steel member, providing a working space for the connection construction. Figure 2b shows a schematic view of the connection between the modules. As a result, the concrete infill is partially encased by the steel member. It is noted that the concrete is filled in the factory except for the connection part of the column.

**Figure 3:**
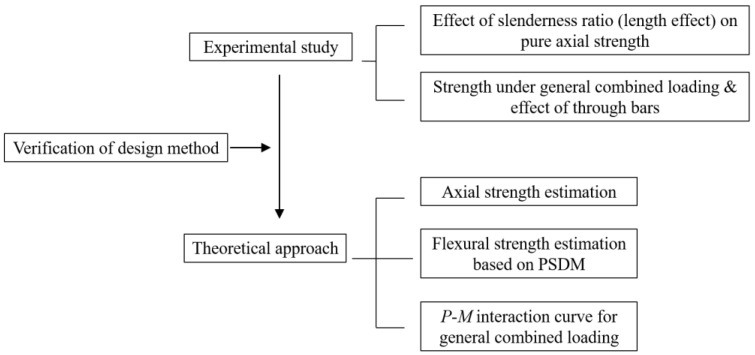
Research flow.

While studies on the nonsymmetrical thin-walled steel (or composite) column have been carried out [17,18,19,20], studies on a nonsymmetrical thin-walled member with concrete infill are limited, and proper design methods are not available due to the geometrical complexity of nonsymmetrical section. Thus, in this study, the strength evaluation and design method development of the proposed composite column for the modular unit structure was investigated through a series of tests and theoretical approaches. Four full-scale specimens were constructed and tested. The main parameters are the slenderness ratio, eccentricity, and the effect of the through bars installed in the steel member to prevent the lateral expansion of the cross section. The design equations and procedure used to evaluate the strength of the proposed composite column were proposed based on the plastic stress distribution method (PSDM) and EC4 (Eurocode 4) [21]. Finally, the proposed design method was verified by comparing it with the test results. The summarized research flow of this study is shown in Figure 3.

## 2. Experimental Study

### 2.1. Description of Test Specimen and Setup

The dimensions of the test specimens and material properties are summarized in Table 1. Four test specimens were constructed with the same cross-section dimensions. The long and short widths of the steel section (*b* and *b*_1_) were 150 mm and 75 mm, respectively (refer to Figure 2a). The thickness of the steel, *t*, was 4.5 mm for all test specimens. The concrete was placed inside the steel member, as shown in Figure 2a. In Table 1, PAL, CL, and TB represent pure axial loading (PAL), combined loading (CL), and through bar (TB), respectively. For example, PAL25 means that the test specimen was under pure axial loading with a length of 2500 mm.

**Figure 4:**
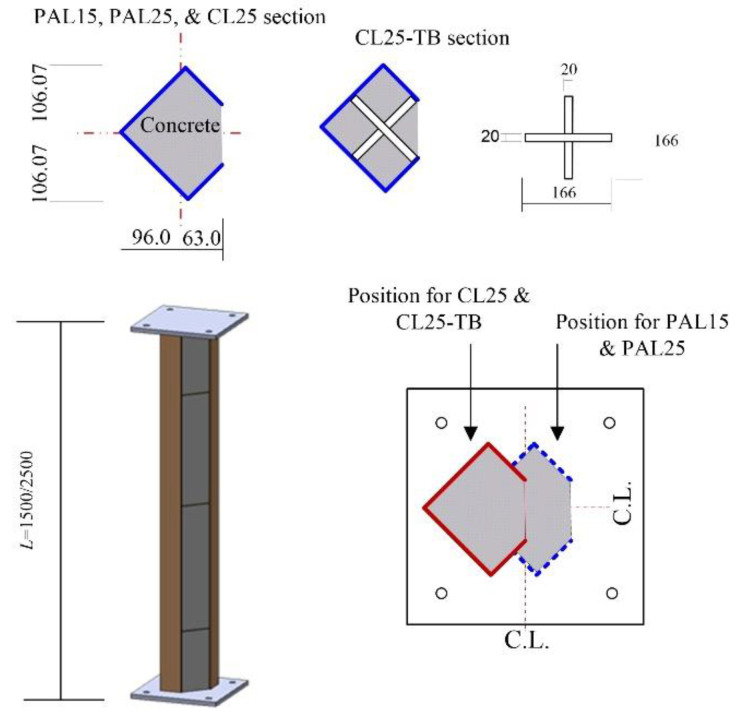
Schematic view of test specimens (unit: mm).

PAL15 and PAL25, described in Table 1, were the test specimens used to examine the length effect, where eccentricity, *e*, was equal to zero. The lengths of PAL15 and PAL25 were 1500 mm and 2500 mm, respectively. The purpose of the CL25 specimen was to investigate the effect of combined compression and bending moment, where *e* is set as 75 mm and the other dimensions were the same as those for PAL25. The dimensions of CL25-TB were the same as those for the CS25 specimen, except for the through bar, as shown in Figure 4. Additional through bars were installed to prevent the expansion of the steel section more effectively for the CL25-TB specimens.

**Figure 5:**
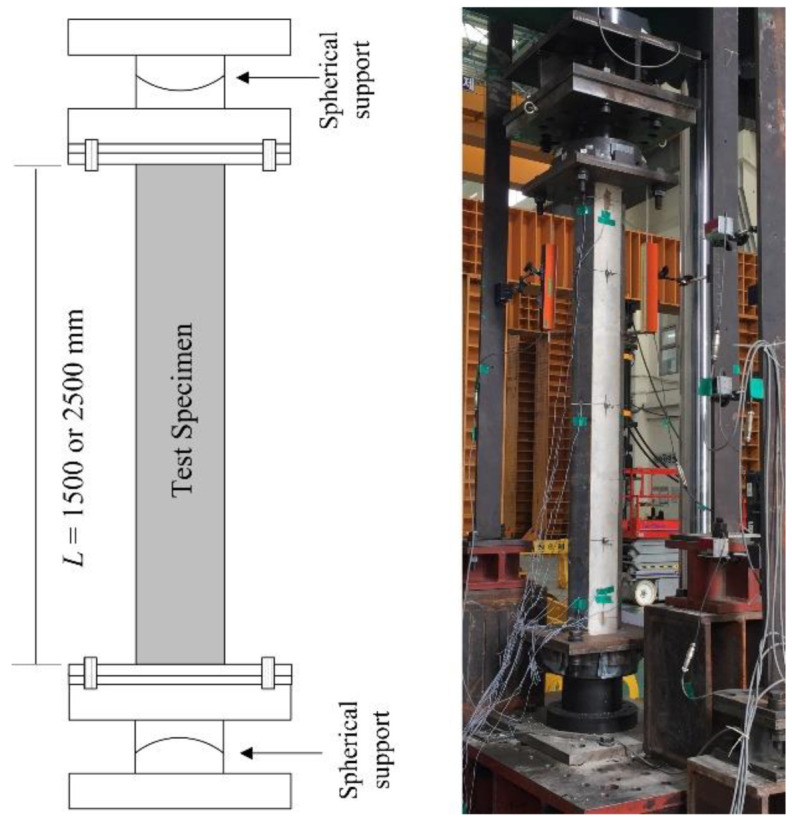
Test setup.

Figure 4 shows a schematic view of a typical test specimen. The diaphragms with a thickness of 4.5 mm were attached at 500 mm spacing for all specimens. The steel plates were welded to both ends of the specimen, as shown in Figure 4. The test specimens were welded with 45° rotation since the section was not symmetrical and the principal axis was 45° rotated about the horizontal axis for the test section. The angle of the principal axis is discussed in detail in Section 3. The welded plates were connected with spherical supports, as shown in Figure 5, to provide a hinge boundary condition. The load was applied via a 2000 kN universal testing machine (UTM). The displacement control was used to capture the post-peak behavior during the test. The speed of loading was 0.01 mm/s.

**Figure 6:**
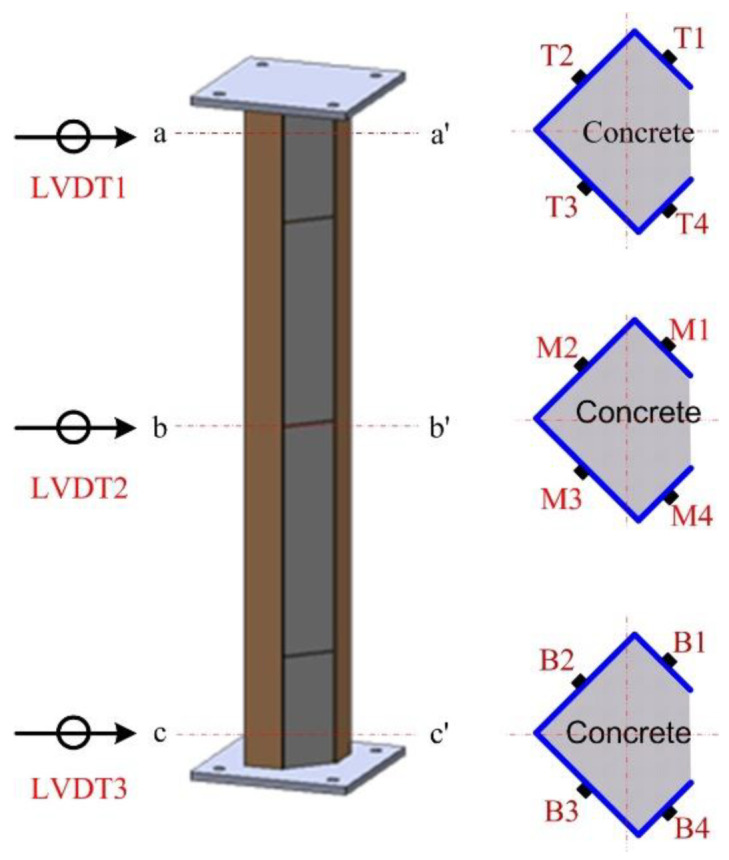
Data acquisition location.

The lateral displacements and strains were measured during the test using linear variable differential transformers (LVDTs) and uniaxial strain gauges. The locations for data measuring are shown in Figure 6. Three LVDTs were used to measure the lateral displacement of the specimens. Vertical displacement was measured using two LVDTs installed at the top and bottom of the specimen. Twelve strain gauges were used to measure the strain in the steel member, as shown in Figure 6.

### 2.2. Test Results

The material test was performed to evaluate the material properties of the test specimens. The compressive strength of the concrete infill, *f_c_*′, and the yield strength of the outside steel member, *f_y_*, were 39.69 MPa and 472.60 MPa, respectively. The modulus of elasticity for the concrete and steel (*E_c_* and *E_s_*) were also evaluated from the material tests. *E_c_* and *E_s_* were 23,975 MPa and 199,090 MPa, respectively.

**Figure 7:**
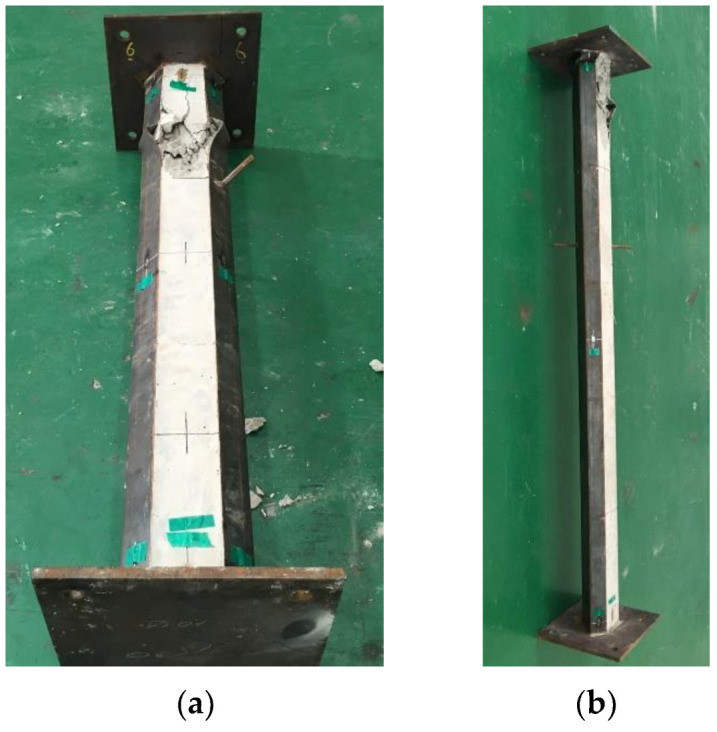
Failure shape: (**a**) PAL15 and (**b**) PAL25.

Figure 7 shows the failure shapes of the PAL15 and PAL25 specimens after the test. For the PAL15 and PAL25 specimens, eccentricity, *e*, was equal to zero and the axial load was applied through the center of gravity of the cross section. The failure mode for both PAL15 and PAL25 was the local buckling of the outside steel member and concrete crushing in the open part of the cross section, as shown in Figure 7a,b. It should be noted that the width-to-thickness ratios for the long and short widths (*b*/*t* and *b*_1_/*t*) were 33.3 and 16.7, respectively. These values satisfied the maximum width-to-thickness ratio specified in EC4 [21]. EC4 specifies the maximum width-to-thickness ratio as 52ε and 44ε for the rectangular hollow section and partially encased section, respectively, where ε is √(235/*f_y_*). One of the edges in the short width (*b*_1_) was free and it was the same as the flange of the partially encased section specified in EC4 [21]. The long width (*b*) can be considered as one of the widths in the rectangular hollow section. Thus, the maximum *b*/*t* and *b*_1_/*t* ratios can be obtained as 52ε and 44ε, respectively according to EC4 [21]. Even when the tested sections had lower *b*/*t* and *b*_1_/*t* ratios than 52ε and 44ε, respectively, local buckling of the steel and concrete crushing occurred at the final stage of the test and the strength was limited by this local failure.

**Figure 8:**
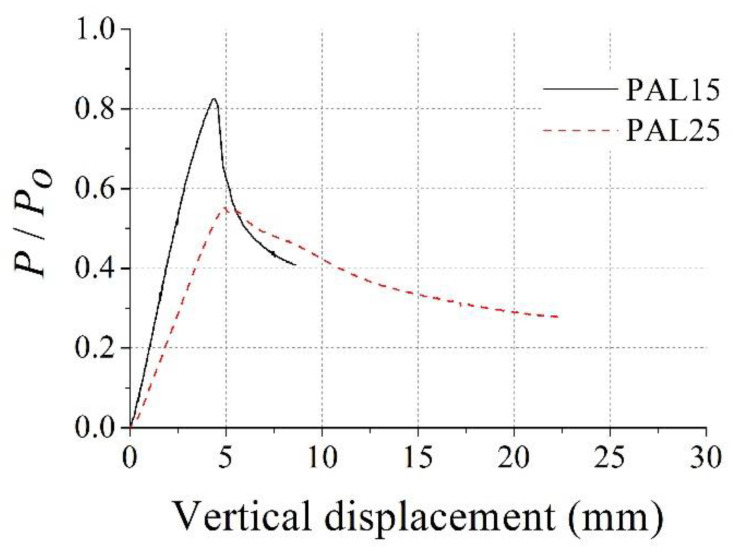
Dimensionless axial load vs. vertical displacement for PAL15 and PAL25.

Global buckling was not observed during the test, since the lateral displacement measured during the test was negligible. The relationships between axial load and vertical displacement are shown in Figure 8, where the *y* axis represents the dimensionless axial load normalized by the squash load, *P_o_*. *P_o_* can be obtained as *f_y_A_s_* + 0.85*f_c_*′*A_c_* [21] and *P_o_* was 1621 kN for the test specimen. As shown in Figure 8, the axial load rapidly reduced after the peak load for both specimens. The maximum *P*/*P_o_* for the PAL15 and PAL25 specimens was 0.83 and 0.55, respectively.

**Figure 9:**
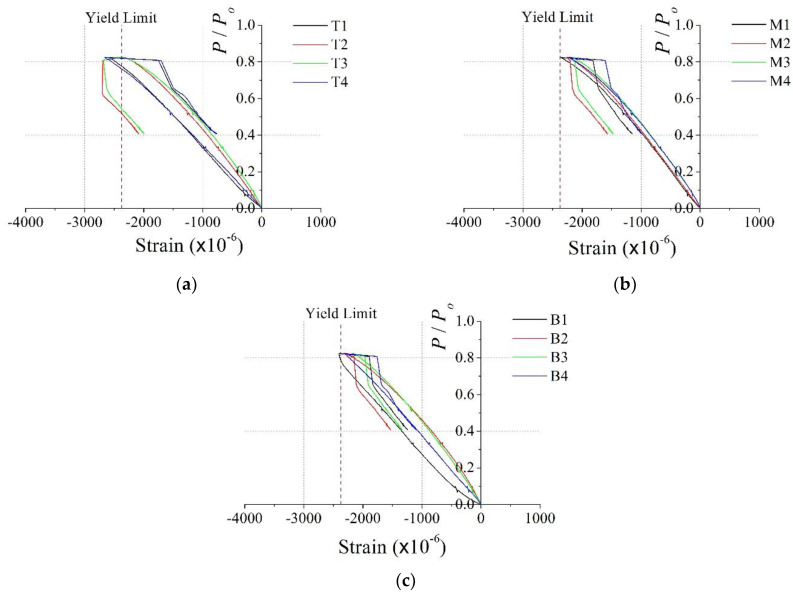
Dimensionless axial load vs. vertical stain for PAL15: (**a**) top (T1–4); (**b**) middle (M1–4); (**c**) bottom (B1–4).

**Figure 10:**
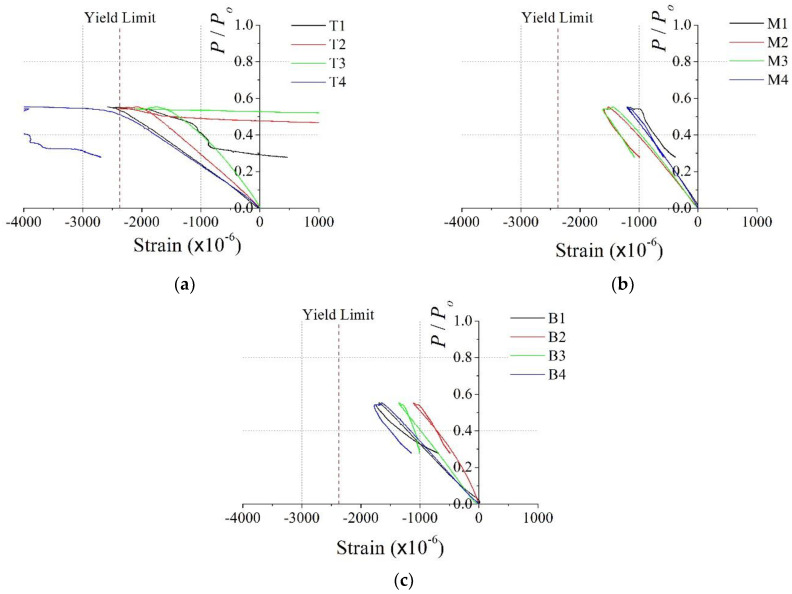
Dimensionless axial load vs. vertical stain for PAL25: (**a**) top (T1–4); (**b**) middle (M1–4); (**c**) bottom (B1–4).

Figure 9 and Figure 10 show the relationship between the dimensionless axial load and the vertical strain in the steel member for the PAL15 and PAL25 specimens, respectively. The location of the strain gages is shown in Figure 6. In Figure 9 and Figure 10, the dashed line represents the uniaxial yield strain of the steel, where the yield strain is calculated as *f_y_*/*E_s_* = 2374 × 10^−6^. Figure 9 illustrates the similarity among the slopes of the strain data. This indicates that almost uniform axial force is developed in the section during the test of the PAL15 specimen. The figure also shows that the almost vertical strains in the steel member reached yield strain at the ultimate state. Thus, yield stresses were developed in the entire steel member simultaneously. However, the strains in the top part of the specimen were somewhat greater than those in the other parts of the specimen, which might lead to early local failure in the upper part of the specimen.

In the case of PAL25, the strains in the bottom and middle sections were similar and did not reach yield strain, as shown in Figure 10b,c. On the other hand, the top strains were much larger than the strains in the bottom and middle sections, and some strains reached yield strain. This implies that the load was concentrated at the top of the test specimen and the bottom and middle sections remained in an elastic state. This is because the load was applied from the top of the specimen, and the length of PAL25 is larger than PAL15. Consequently, the axial load along the length is not uniform and local failure of the upper part occurred first. Additionally, with increasing the length of the specimen, the strain distribution in the specimen became nonuniform, and additional reinforcements such as the through bar inside the steel member were necessary for uniform load transfer.

The dimensions of the CL25 and CL25-TB specimens were the same, except for the existence of the through bar inside the column. The eccentricity, *e*, was 75 mm for both specimens. Thus, a bending moment was generated in the column and could be calculated as *P*(*e* + Δ), where *P* and Δ were the applied axial load and the lateral deflection of the column, respectively.

**Figure 11:**
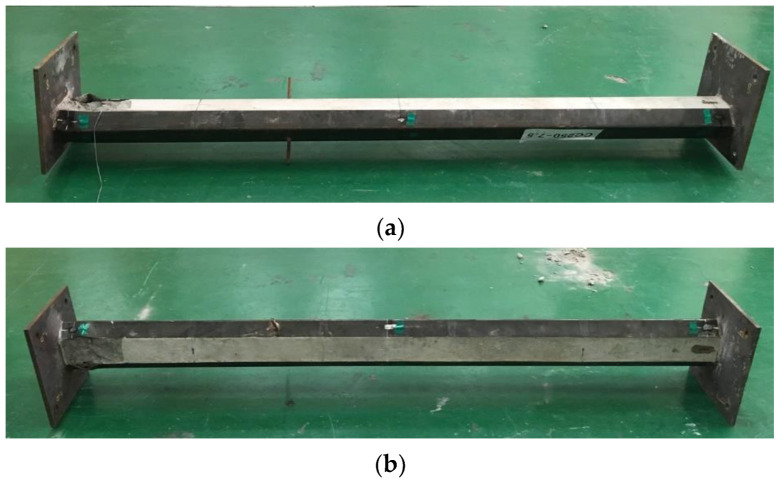
Failure shape: (**a**) CL25; (**b**) CL25-TB.

**Figure 12:**
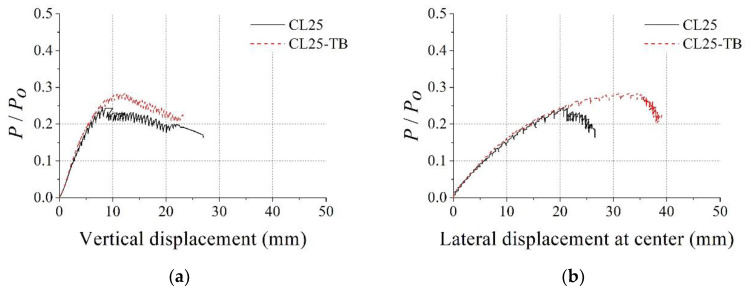
Test results for CL25 and CL25-TB: (**a**) dimensionless axial load vs. vertical displacement; (**b**) dimensionless axial load vs. lateral displacement at the center.

Figure 11a,b show the failure shape of the CL25 and CL25-TB specimens after the test, respectively. Similar to the PAL15 and PAL25 specimens, local buckling of the steel member and concrete crushing were observed at the top of the specimen. However, before local failure, considerable lateral displacement was observed during the tests. Figure 12a,b show *P*/*P_o_* vs. vertical displacement curve and *P*/*P_o_* vs. lateral displacement at the center curve, respectively. The maximum *P*/*P_o_* of CL25 and CL25-TB were 0.25 and 0.28, respectively.

**Figure 13:**
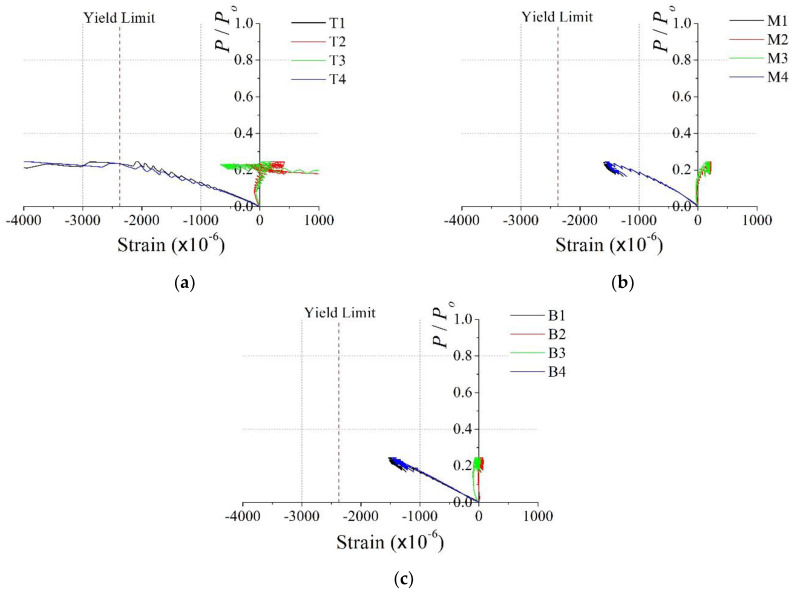
Dimensionless axial load vs. vertical stain for CL25: (**a**) top (T1–4); (**b**) middle (M1–4); (**c**) bottom (B1–4).

**Figure 14:**
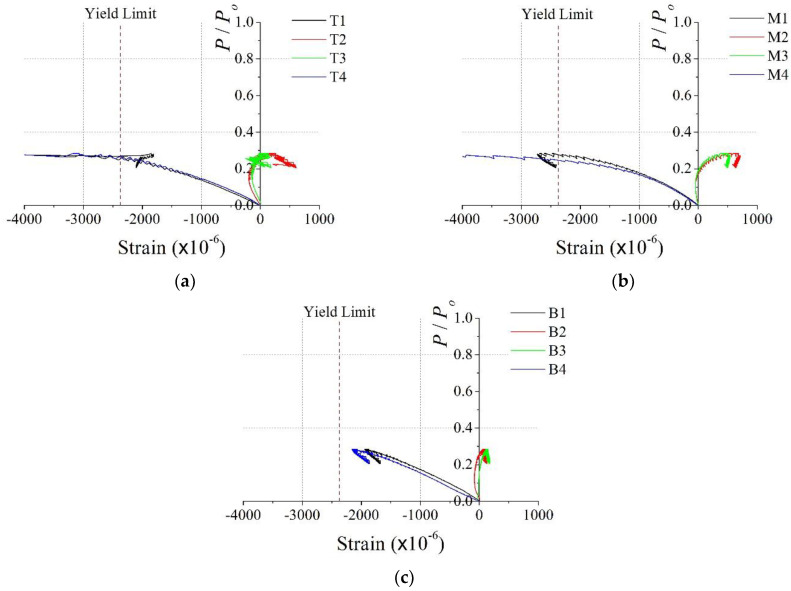
Dimensionless axial load vs. vertical stain for CL25-TB: (**a**) top (T1–4); (**b**) middle (M1–4); and (**c**) bottom (B1–4).

Considerable bending behavior was observed before reaching the maximum axial load, as shown in Figure 12b, for both test specimens. Additionally, the stiffnesses of the CL25 and CL25-TB specimens were almost the same. However, the axial load capacity of CL25-TB was approximately 15% higher than that of the CL25 specimen. This implies that the through bars delayed the local buckling of the steel member and increased the strength of the specimen. As mentioned previously, the *b*/*t* and *b*_1_/*t* ratios of the test section satisfied the EC4 width-to-thickness ratio limit [21]. Thus, the strength of the section must be governed by global behavior. However, the test result shows that the local behavior affected the strength of the test specimen. Thus, for the proposed column section, through bars were needed to delay (or prevent) the local failure, even if the maximum width-to-thickness ratio in EC4 [21] was satisfied. Additionally, through bars helped the uniform distribution of the stresses in the specimens, and this was confirmed by strain data shown in Figure 13 and Figure 14.

Figure 13 and Figure 14 show the relationship between the dimensionless axial load and vertical strains in the steel member for the CL25 and CL25-TB specimens, respectively. Due to the effect of eccentricity, bending moments were generated and the strain distributions significantly differed from those of the PAL15 and PAL25 specimens. The strain distributions in Figure 13 demonstrate that the strains in the top section were larger than those in the bottom and middle sections. This leads to load concentration and the early local failure of the top section of the CL25 specimen.

In the case of the CL25-TB specimen, the strain distributions in the top, middle, and bottom sections were similar, and the compression strains in the top and middle sections reached yield strain. This means that the axial forces and bending moments acting along the length in the CL25-TB specimen were more uniform than the CL25 specimen. Therefore, local failure was delayed, and a larger strength was achieved than that of the CL25 specimen. From the test results of the CL25 and CL25-TB specimens, it was concluded that through bars inside the column were needed for uniform load distribution and delay of local failure of the proposed column section.

## 3. Design Equations

### 3.1. Axial Strength

**Figure 15:**
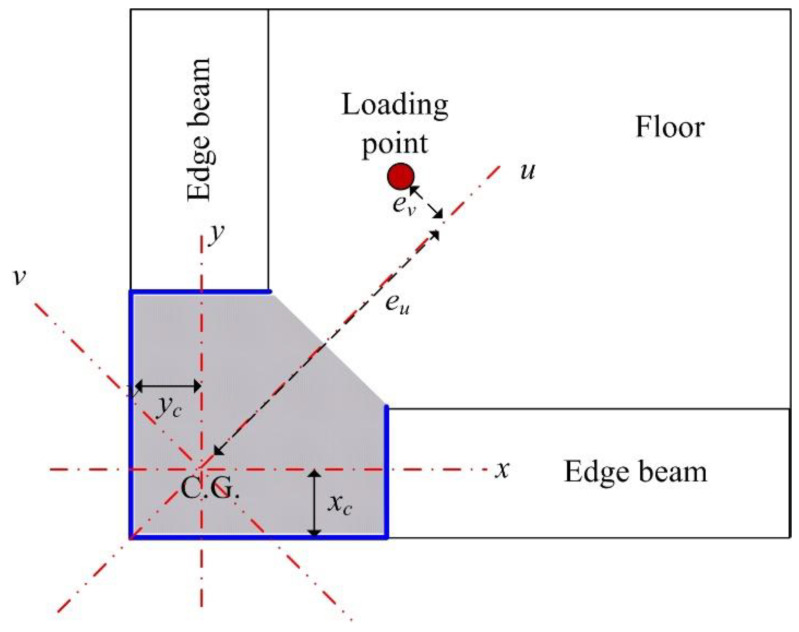
Nonsymmetrical composite column and loading position.

The section of the proposed column was not symmetrical. Thus, biaxial bending and torsional behavior could occur under axial load. However, torsional behavior under axial load was small enough to ignore since the section was concrete filled. In the proposed nonsymmetrical composite column, the principal axis was rotated 45° from the horizontal axis, as shown in Figure 15, where *u* and *v* are the principal axes. Thus, for arbitrary loading having eccentricity *e_u_* and *e_v_*, the applied load can be divided into the axial load at the centroid and the bending moments about the principal axes. For example, in the cases of the CL25 and CL25-TB specimens where *e_u_* and *e_v_* were 75 mm and 0 mm, respectively, only the bending moment about the *v* axis and axial force at the centroid were available. Therefore, this can be treated as a two-dimensional problem.

By assuming that the torsional behavior under axial compression is small and flexural buckling and is the first buckling mode of the proposed column, the classic Euler buckling theory can be applied. In this case, local buckling must be prevented to use this theory. The test results showed that the through bars were necessary to delay (or prevent) the local failure of the proposed column section, even if the maximum width-to-thickness ratio specified in EC4 [21] was satisfied. Hereafter, for the nonsymmetrical column, it was assumed that the section satisfied the maximum width-to-thickness ratio and that it had sufficient through bars in order to apply the design equations and the procedure described in this section.

The principal axis could be conveniently used to avoid the unnecessary moment coupling effect. The location of the principal axis and the effective second moment of inertia about the principal axis were needed to calculate the buckling strength. The centroid of the composite member could be calculated as

(1) $$x_{c}=\frac{E_{s}\sum A_{s}{\bar{x}}_{s}+E_{c}\sum A_{c}{\bar{x}}_{c}}{E_{s}A_{s}+E_{c}A_{c}}\;\mathrm{and}\;y_{c}=\frac{E_{s}\sum A_{s}{\bar{y}}_{s}+E_{c}\sum A_{c}{\bar{y}}_{c}}{E_{s}A_{s}+E_{c}A_{c}}$$

where *x_c_* and *y_c_* are the distance to the centroid in the *x* and *y* direction, respectively. For the test section, *x_c_* and *y_c_* were both 57.84 mm. The location of the principal axis could then be found from the following equation [22]:

(2) $$tan2\theta =\frac{2I_{xy}}{I_{y}-I_{x}}$$

For a given section, *I_x_* and *I_y_* were the same. Thus, from Equation (2), *θ* was equal to 45°. The effective stiffness of the composite section could be obtained as [21]

(3) $$EI_{eff}=E_{s}I_{s,p}+0.6E_{c}I_{c,p}$$

It is important to note that the long-term effect of concrete was not considered in Equation (3). *I_s,p_* and *I_c,p_* in Equation (3) were the second moment of inertia of steel and of concrete members about the principal axis, respectively. After determining the effective stiffness, the elastic buckling strength and buckling parameter could be obtained as

(4) $$P_{cr,e}=\frac{\pi^{2}EI_{eff}}{{\left(kL\right)}^{2}},\;\lambda =\sqrt{\frac{P_{o}}{P_{cr,e}}}$$

where *P_cr,e_* was the elastic critical buckling strength of the composite member, *k* was the effective length factor (where *k* was equal to one for a simply supported member), and *λ* was the bucking parameter. EC3 (Eurocode 3) [23] provides the buckling curve for various imperfection parameters. By using the buckling curve in EC3 with *λ* in Equation (4), the buckling strength could be calculated. The buckling curve in EC3 [23] was given by

(5) $$\frac{P_{cr}}{P_{o}}=\frac{1}{\Phi +\sqrt{\Phi^{2}-\lambda^{2}}}\;\leq \;1\;\mathrm{and}\;\Phi =0.5\left[1+\alpha \left(\lambda -0.2\right)+\lambda^{2}\right]$$

In Equation (5), *P_cr_* was the critical buckling strength and *α* was the imperfection factor, which was 0.21, 0.34, and 0.49 for buckling curves a, b, and c, respectively. In the case of test specimens, *P_cr_*/*P_o_* was calculated as 0.79 and 0.54 for *L* = 1500 mm (for PAL15) and *L* = 2500 mm (for PAL25, CL25, and CL25-TB), respectively, when the buckling curve c was used. It should be noted that EC4 specifies that the buckling curve in EC3 shall be used to evaluate the buckling strength of the composite column.

### 3.2. Flexural Strength

The strain compatibility method (SCM) [24] and plastic stress distribution method (PSDM) [21,25] are generally used to evaluate the flexural strength and interaction curve for the combined axial load and bending moment, where the length effect is not considered. Moon et al. [26] reported that, while PSDM is simpler and easier to handle, the results do not significantly differ to those with SCM. Thus, PSDM was applied to evaluate the flexural strength of the nonsymmetrical composite column section in this study.

The following assumptions apply for the case of PSDM. (1) The strain distribution was linear across the section, and linear elastic and perfectly plastic material behavior is applied. (2) The concrete was crushed at a compressive strain of 0.003 and stress of 0.85*f_c_*′ for an unconfined section with a rectangular stress block. (3) At the time, steel exceeded the yield strain of *f_y_*/*E_s_*. (4) The tensile contribution of the concrete was negligible.

**Figure 16:**
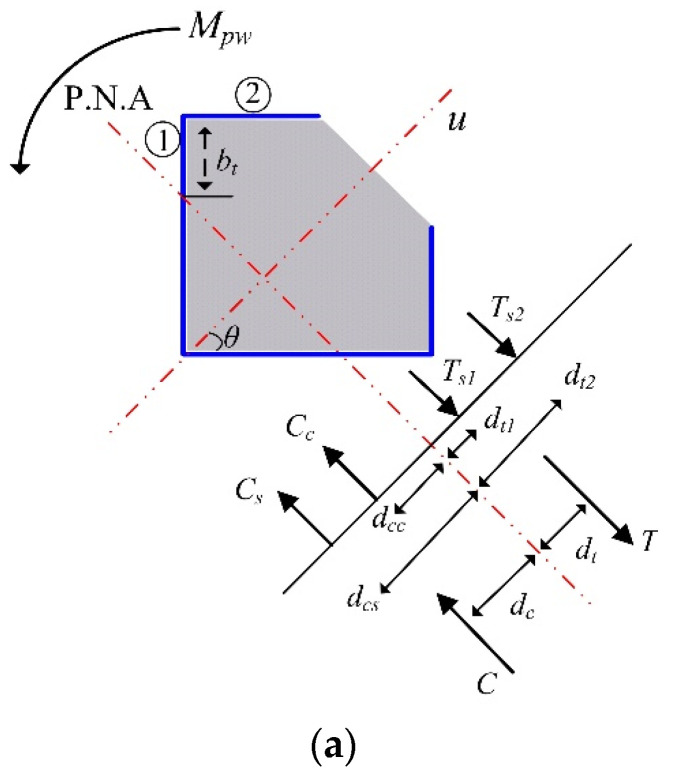

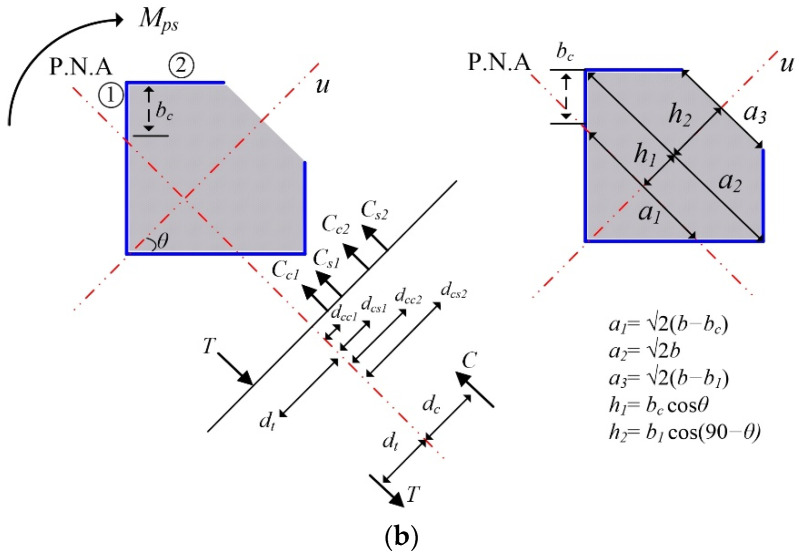
Force distributions in the proposed column section under plastic moment: (**a**) for weak axis; (**b**) for strong axis.

Figure 16 shows an example of plastic moment evaluation for the proposed composite column section. In the case of the test specimens such as CL25 and CL25-TB, *e_v_* was equal to zero and the force distributions shown in Figure 16 can be applied. Two different plastic moments (*M_ps_* and *M_pw_*) can be evaluated. *M_ps_* and *M_pw_* are the plastic moments about the principal *v* axis for strong and weak directions, respectively, as shown in Figure 16.

In Figure 16a, *C_c_* and *C_s_* are the magnitude of compression in the concrete and steel parts, respectively. *T*_*s*1_ and *T*_*s*2_ are the magnitude of tension in the steel member in parts ① and ②, respectively. Then, *C_c_*, *C_s_*, *T*_*s*1_, and *T*_*s*2_ can be calculated as

(6a) $$C_{c}=0.425{\left(b-b_{t}\right)}^{2}f_{c}\prime$$

(6b) $$C_{s}=2\left(b-b_{t}\right)tf_{y}$$

(6c) $$T_{s1}=2b_{t}tf_{y}$$

(6d) $$T_{s2}=2b_{1}tf_{y}$$

The total magnitude of compression, *C*, was equal to *C_c_* + *C_s_*. Similarly, the total magnitude of tension *T* was equal to *T*_*s*1_ + *T*_*s*2_. For a pure bending state, the location of the plastic neutral axis (PNA) can be evaluated from *C* − *T* = 0. For the tested section, PNA was located at *b_t_* = 55.29 mm. After determining the PNA location, the length of the moment arm, *d*, could be calculated as

(7a) $$d=d_{c}+d_{t}$$

(7b) $$d_{c}=\frac{C_{c}d_{cc}+C_{s}d_{cs}}{C}$$

(7c) $$d_{t}=\frac{T_{s1}d_{t1}+T_{s2}d_{t2}}{T}$$

(7d) $$d_{cc}=\frac{1}{3}\left(b-b_{t}\right)\cos\theta =\frac{1}{3\sqrt{2}}\left(b-b_{t}\right)$$

(7e) $$d_{cs}=\frac{1}{2}\left(b-b_{t}\right)\cos\theta =\frac{1}{2\sqrt{2}}\left(b-b_{t}\right)$$

(7f) $$d_{t1}=\frac{1}{2}b_{t}\cos\theta =\frac{1}{2\sqrt{2}}b_{t}$$

(7g) $$d_{t2}=b_{t}\cos\theta +\frac{1}{2}b_{1}\cos\left(90-\theta \right)=\frac{1}{2\sqrt{2}}\left(2b_{t}+b_{1}\right)$$

In Equation (7), *d_c_*, *d_t_*, *d_cc_*, *d_cs_*, *d*_*t*1_, and *d*_*t*2_ were the absolute distances from PNA to *C*, *T*, *C_c_*, *C_s_*, *T*_*s*1_, and *T*_*s*2_, respectively. For the tested section *θ* was 45°. Finally, *M_pw_* could be obtained as *Cd* or *Td*.

In the case of *M_ps_* evaluation, based on Figure 16b, the force components could be calculated as follows:

(8a) $$C=C_{c1}+C_{s1}+C_{c2}+C_{s2}$$

(8b) $$C_{c1}=0.425\left(2bb_{c}-b_{c}^{2}\right)f_{c}\prime$$

(8c) $$C_{s1}=2b_{c}tf_{y}$$

(8d) $$C_{c2}=0.425\left(2bb_{1}-b_{1}^{2}\right)f_{c}\prime$$

(8e) $$C_{s2}=2b_{1}tf_{y}$$

(8f) $$T=2\left(b-b_{c}\right)tf_{y}$$

where *C_c*1*_*, *C*_*s*1_, *C_c*2*_*, and *C*_*s*2_ are the magnitude of compression force in concrete part ①, steel part ①, concrete part ②, and steel part ②, respectively. From *C* − *T* = 0 for the pure bending state, *b_c_* = 2.54 mm for the tested section. The moment arm components in Figure 16b are given by

(9a) $$d=d_{c}+d_{t}$$

(9b) $$d_{c}=\frac{C_{c1}d_{cc1}+C_{s1}d_{cs1}+C_{c2}d_{cc2}+C_{s2}d_{cs2}}{C}$$

(9c) $$d_{cc1}=h_{1}-\frac{h_{1}\left(2a_{1}+a_{2}\right)}{3\left(a_{1}+a_{2}\right)}$$

(9d) $$d_{cs1}=\frac{1}{2}b_{c}\cos\theta =\frac{1}{2\sqrt{2}}b_{c}$$

(9e) $$d_{cc2}=h_{1}+\frac{h_{2}\left(2a_{3}+a_{2}\right)}{3\left(a_{3}+a_{2}\right)}$$

(9f) $$d_{cs2}=b_{c}\cos\theta +\frac{1}{2}b_{1}\cos\left(90-\theta \right)=\frac{1}{2\sqrt{2}}\left(2b_{c}+b_{1}\right)$$

(9g) $$d_{t}=\frac{1}{2}\left(b-b_{c}\right)\cos\theta =\frac{1}{2\sqrt{2}}\left(b-b_{c}\right)$$

In Equation (9), *d*_*cc*1_, *d*_*cs*1_, *d*_*cc*2_, and *d*_*cs*2_ were the absolute distance from PNA to *C*_*c*1_, *C*_*s*1_, *C*_*c*2_, and *C*_*s*2_, respectively. Then, similar to *M_pw_*, *M_ps_* could be calculated as *Cd* or *Td*.

From the calculation results, *M_pw_* and *M_ps_* were 42.39 kN·m and 48.97 kN·m, respectively.

### 3.3. Strength under General Combined Loading

AISC (American Institute of Steel Construction) [25] and EC4 [21] provide the interaction curve under general combined compression and bending moment for a composite column. The interaction curves from two codes can be constructed based on PSDM. The interaction curves from PSDM are then modified by considering the length effect. The approach of two codes to consider the length effect are different each other. In this study, the EC4 [21] method was applied.

**Figure 17:**
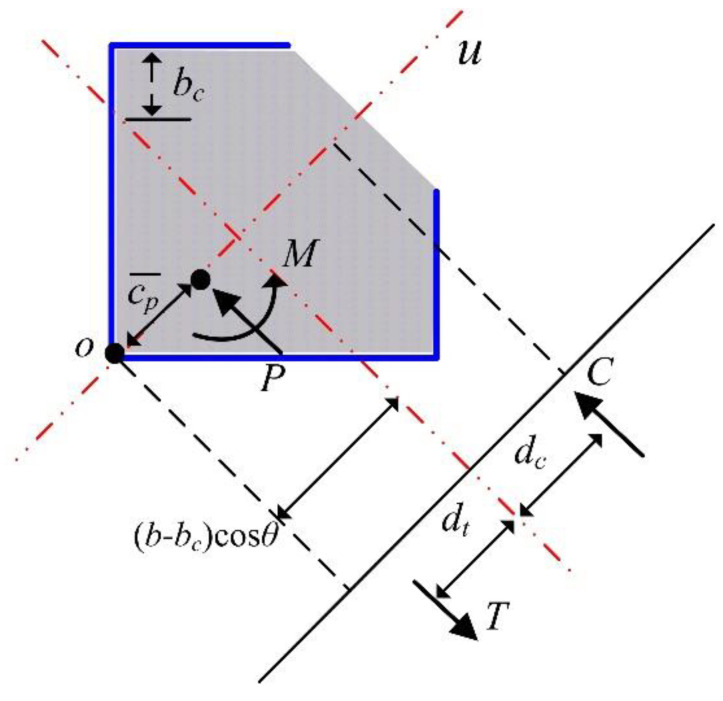
Relationship between *P* and *M* for arbitrary value of *b_c_*.

In Section 3.2, *C* − *T* was equal to zero in order to evaluate the pure flexural strength. In the case of general combined loading, the compression, *P*, may not be zero and *P* could be evaluated as *C* − *T*. Then, the moment of the plastic centroid could be obtained considering the unbalanced compression *P* with eccentricity to the plastic centroid. For the tested section, *e_v_* was equal to zero (i.e., only the bending moment about the principal axis *v* is available. Refer to Figure 15) and the direction of the moment was about the strong axis. Thus, for the arbitrary value of *b_c_*, the relationship between *P* and *M* could be obtained from Figure 17.

From Figure 17, *P* and *M* were calculated as

(10a) P=C−T

(10b) $$M=Td+P\left[\left(b-b_{c}\right)\cos\theta +d_{c}-{\bar{c}}_{p}\right]=Td+P\left[\frac{\left(b-b_{c}\right)}{\sqrt{2}}+d_{c}-{\bar{c}}_{p}\right]$$

In Equation (10), ${\bar{c}}_{p}$ was the distance from *o* to the plastic centroid. It is important to note that Equation (10) is only applicable for a positive value of *b_c_*. By using Equation (10), the interaction curve based on PSDM could be obtained for the tested column.

**Figure 18:**
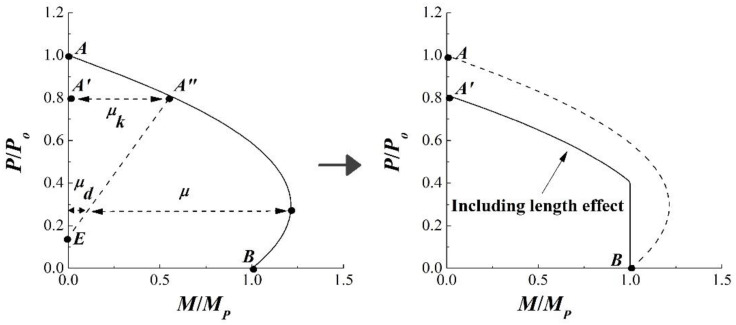
Interaction curve form EC4.

To consider the length effect of the column, the interaction curve based on PSDM must be modified. Figure 18 shows the interaction curve including the length effect from EC4 [21]. In Figure 18, points *A* and *B* represent the full squash load and pure flexural strength, respectively. Point *A* reduced to point *A*′ with the reduction factor, *P_cr_*/*P_o_*. The moment capacity at point *A*′, *μ_k_*, was then removed from the interaction curve and this removal moment decreased linearly to point *E* according to EC4 [21]. By removing *μ_d_* from the interaction curve from PSDM, the interaction curve including the length effect could be constructed. It is important to note that *M*/*M_p_* cannot exceed one when the length effect is considered. In the case of the test specimen, *e_v_* was equal to zero and only the moment about the *v* axis was available. Thus, the interaction curve could be constructed in a two-dimensional (2D) plane, as shown in Figure 18.

## 4. Comparison of Proposed Design Equations with Test Results

**Figure 19:**
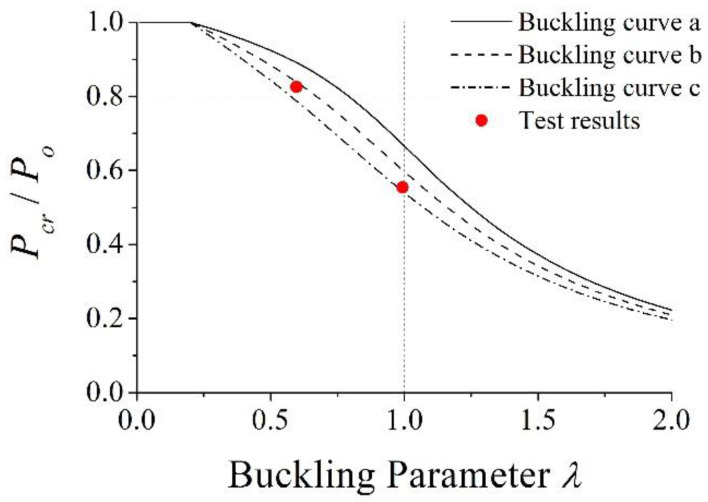
Comparison of the buckling curve from EC3 with test results.

In this section, the strengths of the test specimens obtained from the design procedure described in Section 3 are compared to those obtained from the test results. For PAL15 and PAL25, where only axial loads were applied to the specimen, the theoretical buckling strength, *P_cr_*, could be evaluated from Equation (5). From this calculation, *P_cr_*/*P_o_* were 0.79 and 0.54 for PAL15 and PAL25, respectively, when the buckling curve c was applied. The ratios of the critical load from the test to the squash load, *P_cr,test_*/*P_o_*, were 0.83 and 0.55. The maximum discrepancy was 4% and the average difference was 2.5%. It should be noted that PAL15 and PAL25 did not have through bars inside the column, and local failure occurred. Even if local failure limited the strength of the test specimens, the buckling curve c in EC3 [23] agreed well with the test results, which may provide a conservative prediction of the axial strength of the proposed column with through bars. The comparison results are also shown in Figure 19, again demonstrating that the buckling curve c provides a good prediction of the axial strength of the nonsymmetrical composite column for the unit modular structure.

**Figure 20:**
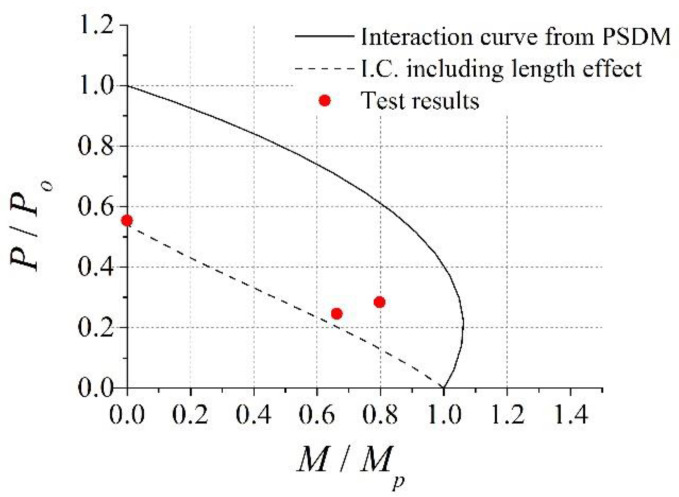
Comparison of the interaction curve from EC4 with the test results [L = 2500 mm].

The interaction curve for the test specimens, where *L* = 2500 mm, was constructed using the procedure described in Section 3.3 and is shown in Figure 20. The solid line and dashed line in Figure 20 represent the interaction curve from PSDM and the interaction curve including the length effect, respectively. The balanced point was observed at approximately *P*/*P_o_* = 0.22. At this point *M*/*M_p_* = 1.06. Only a 6% moment capacity increase was noted at the balanced point. This may be attributed to the high-steel area, *A_s_*, to concrete area, *A_c_*, ratio. For the test specimen, *A_s_*/*A_c_* was 0.10, and it was somewhat larger than other types of composite structure. The interaction curve, including the length effect shown in Figure 20, was constructed with a reduction factor *P_cr_*/*P_o_* = 0.54 and assuming point *E* in Figure 18 was equal to 0.

Figure 20 shows that the interaction curve, when including the length effect, agreed well with the test results of PAL25 and CL25 (where through bars were not installed), while it underestimated the flexural strength of CL25-TB (the specimen with through bars). Thus, it can be concluded that the proposed design equations provided a conservative strength prediction of the nonsymmetrical composite column for unit modular structures.

The design methods suggested in this study were based on the limited test results. It is expected that the design methods could be improved and optimized through a series of additional tests and numerical analyses considering various loading and boundary conditions. Additionally, only theoretical design equations when *e_v_* is equal to 0 were derived to simplify the design condition. The design methods for more general load cases including biaxial bending might be derived by using similar theoretical approaches shown in Section 3. It is beyond the scope of this study and further studies are necessary.

## 5. Conclusions

In this study, the strength of the partially encased nonsymmetrical steel-concrete composite column for the unit modular structure was investigated. For the axial load test without eccentricity (PAL15 and PAL25), the local buckling and concrete crushing were observed at the critical load, even when the steel plate elements in the column satisfied the maximum width-to-thickness ratio. After peak axial load, very sharp strength degradation was observed. In the case of PAL 15, the uniform stress distribution along the length was developed and the steel part reached yield stress. On the other hand, when the length of the specimen was increased (for PAL 25), early local failure at the top of the specimen was observed due to stress concentration at the top of the test specimen.

For the specimens with eccentricity (CL25 and CL25-TB), considerable bending behavior was recorded before failure. Then, local buckling and concrete crushing were observed, reducing the strength. The test results demonstrate that the through bars inside the column delay the local failure and distribute the load more uniformly along the length of the specimen. Thus, through bars were needed for the proposed column section, even when the maximum width-to-thickness ratio specified was satisfied.

The design method for the proposed column was proposed. The axial strength was obtained based on the effective flexural stiffness about the principal axis and the buckling curve c in EC3. The pure flexural strength and *P*–*M* interaction curve, excluding the length effect, were derived from PSDM in closed form solution. The length effect was then applied for the *P*–*M* interaction curve according to EC4. The strengths of the test specimens were evaluated from the proposed design methods and compared with test results. The proposed design methods provide conservative predictions of the proposed composite column for the unit modular structures even if through bars are not installed in the specimen.

## Figures and Tables

**Table 1:** Description of test specimens.

Name	*E* (mm)	*b* (mm)	*b*_1_ (mm)	*t* (mm)	*L* (mm)	Remark
PAL15	0	150	75	4.5	1500	-
PAL25					2500	-
CL25	75				2500	-
CL25-TB					2500	Through bars were installed
